# Seasonal dynamics in the mammalian microbiome between disparate environments

**DOI:** 10.1002/ece3.10692

**Published:** 2023-12-18

**Authors:** Mason R. Stothart, Hayley A. Spina, Michelle Z. Hotchkiss, Winnie Ko, Amy E. M. Newman

**Affiliations:** ^1^ Department of Integrative Biology University of Guelph Guelph Ontario Canada; ^2^ Faculty of Veterinary Medicine University of Calgary Calgary Alberta Canada; ^3^ Department of Biology University of Ottawa Ottawa Ontario Canada

**Keywords:** 16S amplicon, bacteria, grey squirrel, gut, landscape modification, urban, wildlife

## Abstract

Host‐associated bacterial microbiomes can facilitate host acclimation to seasonal environmental change and are hypothesized to help hosts cope with recent anthropogenic environmental perturbations (e.g., landscape modification). However, it is unclear how recurrent and recent forms of environmental change interact to shape variation in the microbiome. The majority of wildlife microbiome research occurs within a single seasonal context. Meanwhile, the few studies of seasonal variation in the microbiome often restrict focus to a single environmental context. By sampling urban and exurban eastern grey squirrel populations in the spring, summer, autumn, and winter, we explored whether seasonal rhythms in the grey squirrel gut microbiome differed across environments using a 16S amplicon sequencing approach. Differences in the microbiome between urban and exurban squirrels persisted across most of the year, which we hypothesize is linked to anthropogenic food consumption, but we also observed similarities in the urban and exurban grey squirrel microbiome during the autumn, which we attribute to engrained seed caching instincts in preparation for the winter. Host behaviour and diet selection may therefore be capable of maintaining similarities in microbiome structure between disparate environments. However, the depletion of an obligate host mucin glycan specialist (*Akkermansia*) during the winter in both urban and exurban squirrels was among the strongest differential abundance patterns we observed. In summary, urban grey squirrels showed different seasonal patterns in their microbiome than squirrels from exurban forests; however, in some instances, host behaviour and physiological responses might be capable of maintaining similar microbiome responses across seasons.

## Graphical abstract

We sampled urban and exurban eastern grey squirrel populations in the spring, summer, autumn, and winter to explore whether seasonal rhythms in the grey squirrel gut microbiome differed across environments using a 16S amplicon sequencing approach. In summary, urban grey squirrels showed different seasonal patterns in their microbiome than squirrels from exurban forests; however, in some instances, host behaviour and physiological responses might be capable of maintaining similar microbiome responses across seasons.

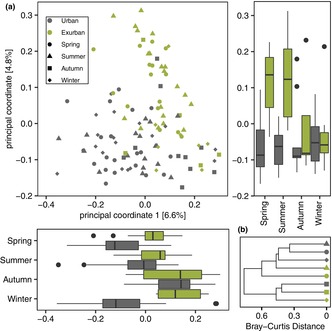

## 1 INTRODUCTION

Gut microbiomes are now understood to play an important role in shaping the niche occupied by their animal host. Host‐associated microbiota unlock otherwise inaccessible dietary nutrients (Han et al., 2022), participate in inter‐specific communication (Sharon et al., 2010), and even tune the physiological and behavioural phenotypes of their hosts (Sudo et al., 2004; Vuong et al., 2017). However, microbiomes are also highly mutable in response to environmental variation. Environmental conditions can directly affect the microbiome by altering host diets (Kartzinel et al., 2019), or by changing the pool of potential colonizing microbiota to which hosts are exposed (Bornbusch et al., 2022). Indirect effects of environmental change—the ways in which environmental conditions affect host physiology—are likewise important, although more commonly overlooked (Stothart et al., 2019).

Changes in the microbiome, which occur in response to predictable (daily, seasonal, migratory) environmental change, might comprise evolved host responses, if alterations in the microbiome are caused by adaptive physiological responses in the host (Regan et al., 2022). Conversely, even when hosts face environmental challenges to which they are not adapted, the microbiome is hypothesized to help facilitate host population persistence by increasing the phenotypic variation on which selection can act (Alberdi et al., 2016). But what happens to the host‐associated microbiome when multiple forms of environmental change overlap?

Seasonal variation is an example of recurrent environmental change, which has selected for a wide array of adaptations in animal populations, and which can cause changes in the animal gut microbiome (Marsh et al., 2022). At low latitudes, wildlife gut microbiomes commonly differ between the wet and dry seasons (Baniel et al., 2021; Grieneisen et al., 2021). These changes are attributed to diet; however, in a large study of 33 sympatric species of herbivore megafauna in Africa, diet explains only 25% of the seasonal turnover observed in the microbiome (Kartzinel et al., 2019), emphasizing the importance of other factors. Seasonal variation can also cause microbiome changes at high latitudes and altitudes (Ren et al., 2017), especially between the climatic extremes of summer and winter (Bird et al., 2019; Drovetski et al., 2019; Ilina et al., 2021; Wang et al., 2020). Diet is suggested as the primary driver of these dynamics in the microbiome (Fan et al., 2022). However, seasonal changes in host diets coincide with transitions in animal reproductive state (Bronson, 2009), as well as abiotic stressors (e.g., thermal challenges in the winter; Ducharme et al., 1989). Thermal challenge and reproductive condition can both cause change in host‐associated microbiomes (Escallón et al., 2019; Sepulveda & Moeller, 2020).

The microbiome does not passively covary with seasonal environmental change. Rather, the microbiome can help hosts to acclimate to changing seasonal conditions and cope with inclement environmental challenges. The microbiome has been shown to partly shape the cold‐tolerant phenotype of their host in both vertebrates and invertebrates (Kokou et al., 2018; Worthmann et al., 2017). However, the strongest evidence for the microbiome's role in host adaptation to thermal challenges is observed in hibernating mammals. In thirteen‐lined ground squirrels (*Ictidomys tridecemlineatus*), ureolytic microbiota in the intestinal tract contribute to protein homeostasis during hibernation (Regan et al., 2022), and a similar mechanism appears to operate in hibernating bears (Barboza et al., 1997). Remarkably, the metabolic phenotype of hibernating bears can even be partially recapitulated in mice, via faecal microbiome transplantation (Sommer et al., 2016). Despite the apparent importance of the microbiome in shaping host responses to seasonal environments, most wildlife microbiome research has been conducted in a single seasonal context.

In contrast to seasons that are a form of predictable recurrent environmental change that wildlife have experienced over millennia, urbanization is an example of recent environmental change affecting wildlife within the last few centuries. Thus, host–microbiome symbioses may not yet be adapted, or may be currently adapting, to the urbanized landscape. Nonetheless, like seasons, urbanization has been shown to affect wildlife microbiomes (Berlow et al., 2021; Murray et al., 2020; Phillips et al., 2018; Stothart & Newman, 2021). Diet alterations are thought to be the primary mechanism underlying urban related change in wildlife microbiomes (Gadau et al., 2019; Sugden et al., 2020); however, even urban tree populations show a distinct microbiome signature (Laforest‐Lapointe et al., 2017). Furthermore, urbanization can expose wildlife to an array of stressors, which alter host physiology and, therefore indirectly, the microbiome (Stothart et al., 2019). Unlike seasonal variation in the microbiome, it is unclear whether urban‐associated change in the microbiome might help wildlife cope with challenges in the human‐built environment. Or whether the host–microbiome relationship has been altered by anthropogenic diets and stressors in urban environments.

Seasonal variation and urbanization are not mutually exclusive forms of environmental change. Yet, few studies on urban wildlife fully encapsulate annual seasonal variation despite research on urban evolutionary ecology gaining traction in recent years (Rivkin et al., 2019). Importantly, the interactions between these two forms of environmental change will not necessarily have additive effects on the host–microbiome relationship. For example, early evidence suggests that season x environment interactions might play a substantive role in shaping organismal responses to urbanization, or the evolutionary trajectory of urban plant and animal populations (Teyssier et al., 2018; Thompson et al., 2016). To fully understand the ecology of host–microbiome symbioses, it is valuable to study host species in the range of seasonal and environmental contexts in which they occur.

Given: (1) evidence that the selective pressures that act on eastern grey squirrels (*Sciurus carolinensis*) in urban environments are season‐dependent (Cosentino & Gibbs, 2022), (2) our previous findings of microbiome resemblances across separate cities and exurban forests (Stothart & Newman, 2021), and (3) suggestions that the microbiome is important for shaping both the dietary niche (Ley et al., 2008) and thermos‐energetics (Sepulveda & Moeller, 2020) of the host, it is worth characterizing how urbanization affects the grey squirrel microbiome across a fuller seasonal context. Many studies of wildlife microbiomes characterize how environmental or seasonal variation affects host‐associated microbiomes, but rarely both simultaneously. In this study, we explored the question of whether seasonal patterns in the grey squirrel microbiome differ across urban and exurban environments. We hypothesized that seasonal rhythms in host–microbiome composition would be affected by environment due to differences in host‐diet. Here, we use faecal microbiomes samples collected from grey squirrels captured in the spring, summer, autumn, and winter to characterize a full seasonal cross‐section of the grey squirrel microbiome, across an urban and a rural forest reference site.

## 2 METHODS

### 2.1 Sampling protocol

Adult eastern grey squirrels were trapped from 2016 to 2018 (Animal Utilization Protocol no. 3506, Wildlife Scientific Collectors Authorization no. 1087323). The main campus of the University of Guelph (43°31052.3300 N, 80°13036.8000 W, Guelph, ON, Canada) was used as our urban site, while the deciduous forests of the ‘rare Charitable Research Reserve’ (43°22052.1700 N, 80°20052.4600 W, Cambridge, ON, Canada) served as our exurban site. Initial site selection involved qualitative assessments sites (e.g., prominence old growth forest versus human‐built impervious surfaces, presence of refuse bins, human foot‐traffic); however, post hoc quantification of normalized difference vegetation index (NDVI) and normalized difference built‐up index (NDBI) estimates (surrounding locations of sample collection) demonstrates clear differences in both the amount of vegetated area (NDVI_exurban_ = 0.426 ± 0.074; NDVI_urban_ = 0.267 ± 0.081) and the footprint of human‐built constructs (NDBI_exurban_ = −0.311 ± 0.024; NDBI_exurban_ = −0.165 ± 0.108) between sites (Rimbach et al., 2022). Both study sites reside on the treaty lands and territory of the Neutral, Haudenosaunee, and the Anishinaabe peoples.

Squirrels were captured using tomahawk Model 102 traps (Tomahawk Live Trap Co., WI, USA). Traps were baited with either peanuts or oat and peanut butter balls and checked every hour from 06:00 a.m to 4:00 p.m. Following capture, squirrels were transferred to a cloth bag until faecal pellets could be collected (approximately 15 min). Sex and reproductive condition were recorded at the time of capture. Reproductive condition was coded as either reproductive female (lactating or in oestrus), non‐reproductive female, scrotal male, or non‐scrotal male. All squirrels were provided with a unique pair of alpha‐numeric ear tags for future recapture identification. Faecal pellets were stored on ice in the field until transfer to storage at −20°C. In total, we collected and sequenced faecal samples from 112 unique individuals, to avoid pseudo‐replication at the individual level. We collected a total of 64 samples from urban squirrels (*n*_spring_: 20, *n*_summer_: 17, *n*_autumn_: 10, *n*_winter_: 17), 27 of which were from males (*n*_scrotal_ = 21; *n*_non‐scrotal_ = 6) and 37 were from females (*n*_reproductive_ = 7; *n*_non‐reproductive_ = 30). At our exurban site, we collected a total of 48 samples (*n*_spring_: 15, *n*_summer_: 20, *n*_autumn_: 5, *n*_winter_: 8), 27 of which were from males (*n*_scrotal_ = 22; *n*_non‐scrotal_ = 5) and 21 were from females (*n*_reproductive_ = 3; *n*_non‐reproductive_ = 18; Table S1).

### 2.2 Sequencing and bioinformatics

We extracted DNA from 0.2 g of faeces using QIAamp DNA Stool Mini Kits (Qiagen, Hilden Germany). The v4 region of the 16S rRNA gene was amplified in triplicate (primers, 515F: 5′‐GTGYCAGCMGCCGCGGTAA‐3′; 806R: 5′‐GGACTACNVGGGTWTCTAAT‐3′) at MetaGenomBio Inc. (Waterloo, Canada; Walters et al., 2015). Triplicate PCR products were pooled and sequenced to a depth of 30,000 reads/sample on an Illumina MiSeq platform (v2 chemistry). Briefly, we merged paired‐end reads and performed quality filtering using a standardized mothur pipeline (Kozich et al., 2013). Paired‐end reads were clustered to operational taxonomic units (OTUs) based on a 97% 16S rRNA gene sequence similarity using the OptiClust algorithm (Westcott & Schloss, 2017). Taxonomy was assigned to representative v4 sequences using the SILVA v138 reference database (Quast et al., 2013; Yilmaz et al., 2014). Only OTUs, which were present at a relative abundance of 0.001 in at least a single sample, were retained for analysis to remove singletons and potential sequencing errors. OTU counts were rarefied to the lowest read‐count in the dataset (3326 reads) prior to all analyses (Figure S1).

### 2.3 Statistical methods

We estimated both OTU richness (No. of observed OTUs) and evenness (Shannon diversity; Hill et al., 2003) measures of microbiome alpha diversity. To estimate OTU richness and Shannon diversity while accounting for unequal sequencing depths and unobserved taxa, we used the R packages ‘breakaway’ (Willis & Martin, 2015) and ‘DivNet’ (Willis & Martin, 2022), respectively. We then used the betta() function from the ‘breakaway’ package to test for differences in alpha diversity estimates between seasons, environments, or squirrels of differing sex and reproductive condition (Willis et al., 2017). Base cases that were on conditions were varied where necessary, to allow for a comprehensive set of condition pairwise comparisons.

To determine environmental effects on gut‐microbiome β‐diversity, we used permutational multivariate analysis of variance (PERMANOVA; adonis2() function). We analysed four β‐diversity metrics, which were based on OTU presence–absence (Jaccard distance; Jaccard, 1912), OTU relative abundance (Bray–Curtis distance; Bray & Curtis, 1957), the phylogenetic weighting of unshared OTUs (unweighted UniFrac distance; Lozupone & Knight, 2005), and phylogeny and relative abundance‐weighted β‐diversity (weighted UniFrac; Lozupone et al., 2007). Models contained each variable in the following order: sex, reproductive condition, environment, season, and the interaction between environment and season. We performed post hoc pairwise testing (pairwise.adonis2() function; ‘pairwiseAdonis’ package: Martinez Arbizu, 2020), in which we compared Bray–Curtis distance across environments within each season, and across seasons within each environment.

Statistical tools for testing for differential abundance in microbiome datasets can yield inconsistent and sometimes spurious results (Nearing et al., 2022). Therefore, it has been recommended that researchers focus on results, which are concordant between different statistical tests. To explore the differential abundance of bacterial genera across seasons and environments, we used a combination of Analysis of Compositions of Microbiomes with Bias Correction (ANCOM‐BC; Lin & Peddada, 2020) and ANOVA‐Like Differential Gene Expression 2 (ALDEx2; Fernandes et al., 2013) tests, which have been identified as the most reliable differential abundance testing methods (Nearing et al., 2022). Among ANCOM‐BC tests, we used a conservative error estimator and applied a false discovery rate correction. Among ALDEx2 tests, we used Benjamini–Hochberg corrected p‐values from Wilcoxon rank sum tests to determine significance. OTUs were binned to genera for differential abundance testing. OTUs, which could not be classified to a genus, were binned to the finest taxonomic resolution, which could be identified.

## 3 RESULTS

### 3.1 Microbiome composition

After filtering and rarefying the data, we observed a total of 1060 OTUs. The most abundant families were the same across seasons and environments, although the proportions varied slightly. The most abundant families, accounting for ~70% of all reads, were as follows: *Lachnospiraceae* (39% ± 13% SE), *Muribaculaceae* (10% ± 8% SE), *Prevotellaceae* (9% ± 7% SE), *Oscillospiraceae* (7% ± 5% SE), and *Ruminococcaceae* (6% ± 5% SE). The average squirrel microbiome had 525 OTUs ±9 SE.

### 3.2 Alpha diversity

Estimated OTU richness was greater in males than females (32 ± 11 SE OTUs, *p* = .003), but did not differ with reproductive state (*p* = .43), and we observed no evidence for an interaction between sex and reproductive state (*p* = .49). The microbiomes of urban squirrels were more diverse than exurban squirrel microbiomes (20 ± 10 SE OTUs, *p* = .03). Squirrels had more diverse microbiomes in the summer than squirrels sampled in the autumn (73 ± 22 SE OTUs, *p* = .001) or winter (62 ± 16 SE OTUs, *p* < .001), but not the spring (*p* = .68). Squirrels likewise had more diverse microbiomes in the spring than in the autumn (79 ± 22 SE OTUs, *p* < .001) or winter (67 ± 22 SE OTUs, *p* < .001). No difference in OTU richness was observed between samples collected in the autumn versus winter (*p* = .61; Figure S2).

Based on estimates of Shannon diversity, males had microbiomes that were more evenly structured than females (0.23 ± 0.05 SE, *p* < .001). Reproductive condition in both males (scrotal) and females (oestrus or lactating) was associated with a decrease in Shannon diversity (−0.19 ± 0.05 SE, *p* < .001), and a significant interaction was observed, whereby this decrease was larger among males (0.13 ± 0.06 SE, *p* = .02). No difference in Shannon diversity was observed between squirrels at urban versus exurban sites (*p* = .2). Likewise, samples collected in the summer were no more even in their composition than those collected in the autumn (*p* = .78) or winter (*p* = .78), but had lower Shannon diversity than samples collected in the spring (0.14 ± 0.06 SE, *p* = .02). No difference in Shannon diversity was observed between samples collected in the spring versus autumn (*p* = .21), spring versus winter (*p* = .09), nor the winter and autumn (*p* = .95).

### 3.3 β‐diversity

**FIGURE 1:**
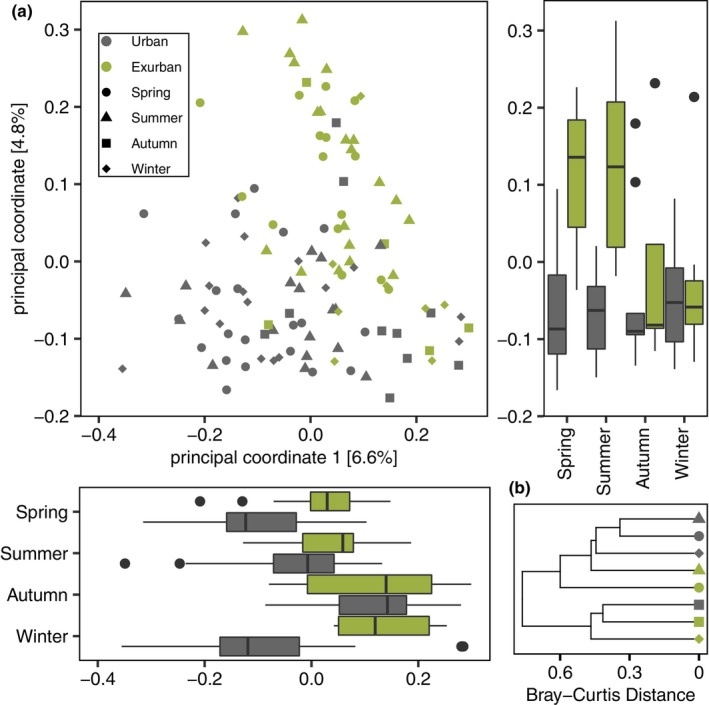
(a) Principal coordinate plot of Bray–Curtis dissimilarities in the eastern grey squirrel faecal microbiome, with the first and second principal coordinates of ordinations visualized in boxplots separated by season and environment and (b) a hierarchical clustering plot of Bray–Curtis dissimilarities from rarefied samples pooled by season and environment, using a ward D method.

We observed effects of sex, reproductive state, environment (urban versus exurban), and season on Bray–Curtis, Jaccard, weighted UniFrac, and unweighted UniFrac dissimilarities (Table 1). A significant interaction between season and environment was also observed with respect to Bray–Curtis, Jaccard, and unweighted UniFrac dissimilarities. To investigate these interactions, we performed post hoc comparisons using the *pairwiseAdonis*() function. In the full dataset, significant differences in Bray–Curtis dissimilarities were observed between all season pairwise comparisons (Table S2). Statistically significant inter‐seasonal differences were also observed among urban squirrels (Table S3), but no difference was observed between samples collected in the autumn and winter from exurban squirrels (*F* = 1.10, *p* = .28; Table S4). Significant differences between urban and exurban squirrels were observed in the summer (*R* ^2^ = .06, *F* = 2.34, *p* < .01), spring (*R* ^2^ = .08, *F* = 2.71, *p* < .01), and winter (*R* ^2^ = .08, *F* = 2.01, *p* < .01), but not in the autumn (*R* ^2^ = .07, *F* = 1.01, *p* = .40). Despite an apparent effect of urbanization on the squirrel microbiome (Figure 1a), samples collected from urban squirrels in the autumn were most similar to samples collected from exurban squirrels in the autumn or winter than to other urban samples (Figure 1b). Conversely, samples collected from urban squirrels in the spring, summer, and winter were more similar to exurban samples collected in the spring or summer than they were to urban samples collected in the autumn.

**TABLE 1:** Results of PERMANOVA tests of physiological and environmental factors that effect variation in the eastern grey squirrel microbiome.

Term	df	*R* ^2^	*p* value
**Bray–Curtis**			
Sex	1	.01	.008*
Reproductive Condition	3	.03	.001*
Environment	1	.03	.001*
Season	3	.06	.001*
Season × Environment	3	.03	.006*
**Jaccard**			
Sex	1	.01	.013*
Reproductive Condition	3	.02	.001*
Environment	1	.02	.001*
Season	3	.05	.001*
Season × Environment	3	.03	.005*
**Weighted UniFrac**			
Sex	1	.01	.018*
Reproductive Condition	3	.03	.012*
Environment	1	.03	.001*
Season	3	.06	.001*
Season × Environment	3	.03	.08
**Unweighted UniFrac**			
Sex	1	.01	.025*
Reproductive Condition	3	.02	.025*
Environment	1	.04	.001*
Season	3	.05	.001*
Season × Environment	3	.03	.002*

* Denotes significance at *p* < .05.

### 3.4 Differential abundance testing

**FIGURE 2:**
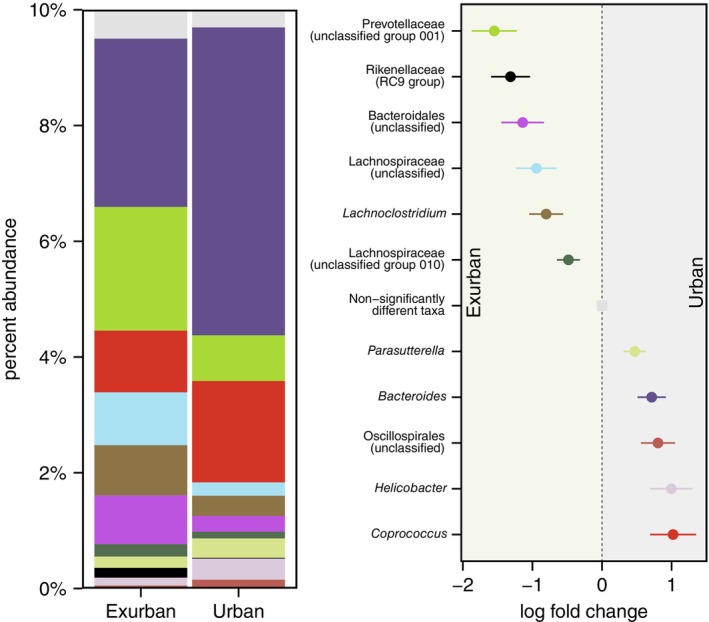
Stacked bar plot of the average per cent abundance of taxa and a forest plot legend of the log fold change in taxon abundance for taxa which significantly differed between the faecal microbiome of eastern grey squirrels sampled from an urban and exurban site in both ANCOM‐BC and ALDEx2 tests. Bars represent standard error in taxon difference estimates from ANCOM‐BC tests. Samples from all seasons pooled by environment type.

**FIGURE 3:**
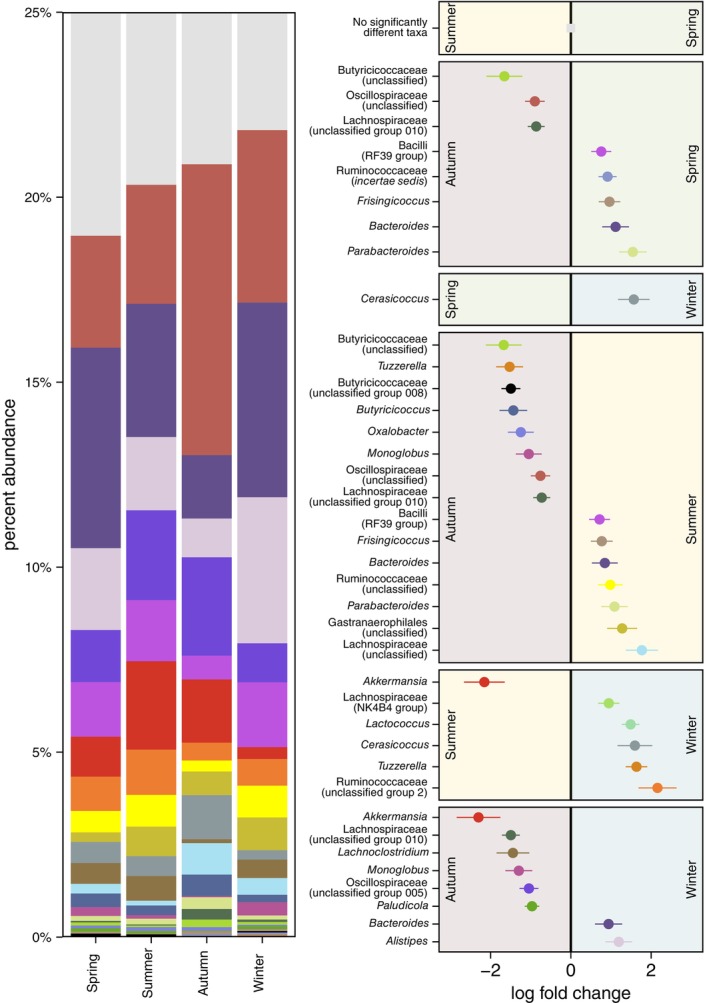
Stacked bar plot of the average per cent abundance of taxa and a forest plot legend of the log fold change in taxon abundance for taxa in the eastern grey squirrel faecal microbiome which significantly differed between seasons in both ANCOM‐BC and ALDEx2 tests. Error bars represent standard error in taxon difference estimates from ANCOM‐BC tests. Samples from urban and exurban environments pooled by season.

Within our full dataset, 11 bacterial taxa were identified by both ANCOM‐BC tests and ALDEx2 to significantly differ in relative abundance between urban and exurban environments (Figure 2). Across the intra‐annual cycle, 24 taxa (*spring v. summer*: 0 taxa, *spring v. autumn*: 8, *spring v. winter*: 1, *summer v. autumn*: 15, *summer v. winter*: 6, *autumn v. winter*: 8) were identified by both ANCOM‐BC tests and ALDEx2 to significantly differ between seasons (Figure 3). See supplemental materials for test outputs (Appendix S2).

**FIGURE 4:**
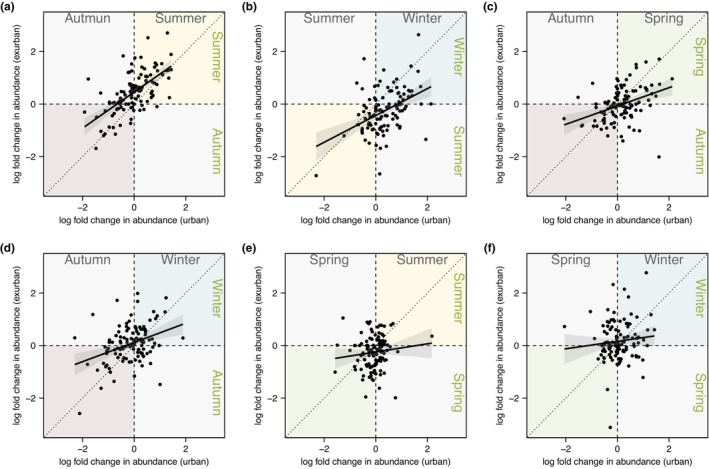
Scatterplots of urban versus exurban ANCOM‐BC estimated log fold change in genus‐level taxon abundances within the eastern grey squirrel faecal microbiome between (a) autumn and summer, (b) summer and winter, (c) autumn and spring, (d) autumn and winter, (e) spring and summer, and (f) spring and winter. Dotted lines denote 1:1 lines. Solid lines denote lines of best fit with 95% confidence interval shading.

To characterize (dis)similarities in the response of the squirrel microbiome to seasonal variation in an urban versus exurban environment, we used ANCOM‐BC tests to estimate taxon log fold changes for separate datasets of urban and exurban squirrels. Urban and exurban estimates of taxon log differences were correlated in the summer versus autumn (*R* ^2^ = .39, *t* = 8.519, *p* < .01; Figure 4a), summer versus winter (*R* ^2^ = .19, *t* = 5.172, *p* < .01; Figure 4b), spring versus autumn (*R* ^2^ = .14, *t* = 4.262, *p* < .01; Figure 4c), and autumn versus winter (*R* ^2^ = .12, *t* = 4.014, *p* < .01; Figure 4d). However, differential abundance estimates were not significantly correlated in the spring versus summer (*p* = .22; Figure 4e) or in the spring versus winter (*p* = .29; Figure 4f) between urban and exurban environments.

## 4 DISCUSSION

We observed seasonal differences in the eastern grey squirrel microbiome, but the magnitude of these differences depended on the seasons being compared. Few microbiome differences were observed between the spring and summer. Conversely, the most apparent seasonal differences in the microbiome occurred in the autumn, relative to the spring or summer. Relative to the spring or summer, squirrels sampled in the autumn had greater relative abundances genera and families, which contain butyrate‐producing bacteria, including Butryicoccaceae, Butyricoccus (Trachsel et al., 2018), a sub‐group 10 of Lachnospriaceae (Meehan & Beiko, 2014), and Oscillispiraceae (Gophna et al., 2017). This may be caused by a dietary shift to acorns and other tree nuts by grey squirrels in the autumn (Koprowski, 1991; Spritzer, 2002). In humans, dietary supplementation with tree nuts has been shown to increase butyrate‐producing bacteria in the gut microbiome (Lamuel‐Raventos & St Onge, 2017; Mandalari et al., 2008; Ukhanova et al., 2014). The ability of tree nuts to enhance butyrate‐producing bacteria growth appears to derive from their fats and oils, since the removal of these compounds fails to replicate the same microbiome patterns seen in response to untreated tree nuts (Mandalari et al., 2008). In addition to nutritional fats and oils, nuts can be high in anti‐nutrient plant‐secondary compounds (PSCs), including tannins and oxalate (Johnson et al., 1993; Massey, 2007). These PSCs could be responsible for the greater abundance of *Oxalobacter* we observed in the autumn relative to the summer, since this genus specializes in the metabolism of plant‐secondary compounds in the mammalian gut (Miller et al., 2016).

Relative to the autumn, squirrels sampled in the spring and summer had greater relative abundances of *Frisingicoccus* (a genus of *Lachnospiraceae*), and unclassified genera of the families *Ruminococcaceae* and *Lachnospiraceae*. *Ruminococcaceae* is a family comprised of dedicated plant fibre degraders (Moraïs & Mizrahi, 2019), while *Lachnospiraceae* are more commonly linked to saccharide fermentation and short‐chain fatty acid synthesis—most notably butyrate (Sorbara et al., 2020). However, butyrate synthesis is present in less than half of Lachnospiraceae (Meehan & Beiko, 2014) and has not been observed in *Frisingicoccus* (Lagkouvardos et al., 2016). The enrichment of these taxa in the spring and summer could reflect a grey squirrel reliance on fresh plant materials at this time of year, including berries, buds, tender leaves, immature maple keys, and pine seeds (Koprowski, 1991; Spritzer, 2002). Squirrels in the spring and summer also harboured greater relative abundances of *Bacteroides* and *Parabacteroides* than in the autumn. These genera are generalists, with members capable of degrading various plant polysaccharides (Wexler, 2007), host glycans, or proteins in an animal‐based diet or host‐derived compounds (David et al., 2014). A generalist microbial niche might be advantageous during the summer and spring when grey squirrels have access to the widest array of dietary items (Nixon et al., 1968).

The grey squirrel microbiome also differed between the winter and autumn, but this divide was characterized by a different subset of taxa, compared to the spring and summer. *Alistipes* and *Bacteroides* were more relatively abundant in the winter than the autumn. Both are bile‐acid tolerant genera associated with the consumption of high‐fat or animal‐based diets (David et al., 2014; Walker et al., 2017). The enrichment of *Alistipes* and *Bacteroides* may reflect a reliance of grey squirrels on animal protein during the winter. Alternatively, microbiota that are able to metabolize host compounds (e.g., *Alistipes* and *Bacteroides*; Sonnenburg et al., 2005) may be relatively unaffected as food becomes limited in the winter, when compared to taxa which are obligately reliant on digesta as a source of nutrients. Interestingly, *Alistipes* show a sixfold increase during the seasonal transition from summer to winter in thirteen‐lined ground squirrels (*Ictidomys tridecemlineatus*), in which they are thought to play a critical role in nitrogen recycling via ureolysis during hibernation (Regan et al., 2022). Although grey squirrels do not hibernate, nitrogen recycling via ureolytic microbiota could still be important for protein homeostasis in the winter when food is limited.

*Akkermanisa* exhibited the greatest seasonal differences and was more abundant in squirrels during the autumn and summer than the winter. *Akkermansia* is a genus of obligate mucin glycan degraders, and so are not reliant on components of the host's diet (Karcher et al., 2021). Chronic stress can cause intestinal inflammation and the depletion of intestinal mucus layers (Gao et al., 2018), and we have previously demonstrated a negative correlation between hair cortisol (a measure of chronic HPA axis upregulation) and *Akkermansiaceae* in the grey squirrel microbiome (Stothart et al., 2019). The presence of *Akkermansia* at greater average abundances in the autumn and summer than the winter might signal greater intestinal mucus production. Conversely, in the winter, thermo‐energetic demands and food depravation might represent stressors of sufficient severity to disrupt mucus barriers in the grey squirrel intestinal tract. The opposite pattern (i.e., an increase in *Akkermansiaceae*) has been observed in ground squirrels during the winter, which upregulate intestinal mucus production during hibernation (Dill‐McFarland et al., 2014).

Seasonal changes in the grey squirrel microbiome between our urban and exurban sites were not always concordant, but urban and exurban squirrel microbiomes were most similar in the autumn. For example, we did not observe a significant effect of urbanization on squirrel microbiome beta‐diversity among samples collected in the autumn. Furthermore, across seasonal comparisons (autumn versus summer/spring/winter), we observed positive correlations in taxon differential abundance estimates made using separate urban and exurban data subsets (Figure 4), indicating similar responses in the microbiome to seasonal differences in these environments. Resemblance in the urban and exurban microbiome in the autumn is evident across the first two principal coordinates in our ordination analysis (Figure 1a) and in the positioning of pooled urban and exurban samples in hierarchical clustering analysis results (Figure 1b). Caching behaviour may play an important role in explaining homogenization of the microbiome. Despite access to anthropogenic food sources, urban grey squirrels spend an appreciable amount of time caching natural food items in the autumn (Thompson, 1980). The engrained instinct to cache and consume tree seeds and nuts in the autumn may preclude the use of anthropogenic food resources, and thus produce similar gut‐microbiome profiles between urban and exurban grey squirrels. In addition to dietary influences, microbiome similarities during the autumn might partly reflect the metabolic changes grey squirrels undergo while acclimating to declining ambient temperatures (Ducharme et al., 1989).

Urban and exurban squirrels displayed different microbiomes in the winter, despite a shared exposure to cold ambient temperatures. Generally, exurban squirrel microbiomes in the winter were more akin to that of the autumn squirrel microbiome, while the microbiomes of urban squirrels in the winter were more alike urban squirrels sampled in the summer and spring. In urban environments, squirrels may return to a reliance on anthropogenic food sources during the winter, when natural food sources are scarce. In contrast, exurban squirrels may be solely reliant on cached seeds or nuts (Nixon et al., 1968). Despite some differences, the depletion of *Akkermansia* (a mucus glycan specialist) during the winter was observed in both urban and exurban squirrels. Therefore, despite potential dietary differences, urban and exurban squirrels may exhibit similar physiological responses to winter conditions.

The largest discrepancy in the response of urban versus exurban squirrel microbiomes to seasonal change was observed between the autumn and spring. Namely, the microbiomes of exurban squirrels tended to be more similar between the spring and autumn than the same seasonal comparison made between urban squirrels, as evidenced by the differing magnitude of PERMANOVA test statistics between pairwise tests (*F*_exurban_ = 1.55; *F*_urban_ = 2.73) and the fewer taxa identified to differ between the spring and autumn in exurban versus urban squirrels (*n*_exurban_ = 10; *n*_urban_ = 20). In contrast, while 17 taxa were identified by ANCOM‐BC tests to differ in abundance between the summer and spring among exurban grey squirrels, only two taxa were identified to differ among urban squirrels during the same seasonal transition. Again, in exurban environments, squirrels may be reliant on cached nuts or during the spring, thereby causing similarity to the autumn squirrel microbiome. The comparably larger differences observed between the spring and summer in exurban squirrels (as compared to urban squirrels) may correspond to small dietary shifts from leaf buds and flowers in the spring, to fruits and seeds in the summer (Moller, 1983). Meanwhile, the reliance of urban squirrels on anthropogenic sources of food—rather than seasonal availability of plant products—might explain the weaker evidence for microbiome differences between spring and summer at our urban site.

In addition to seasonal changes in the grey squirrel bacterial microbiome, we also observed a persistent ‘urban’ microbiome signature. The most appreciable difference we observed was a higher relative abundance of *Bacteroides* but lower relative abundance of unclassified *Prevotellaceae* among urban squirrels compared to exurban squirrels. This is consistent with our own previous multi‐city comparisons of urban and exurban squirrels (Stothart & Newman, 2021), and with the findings from a review of the effects of urbanization on human microbiomes (Zuo et al., 2018). Urban squirrels also had greater relative abundances of *Helicobacter*, unclassified Oscillospirales, and *Parasutterella*, bile‐acid tolerant taxa, which are often enriched in hosts consuming diets rich in animal fats and proteins (Colston & Jackson, 2016; McFarlane et al., 2021; Moon et al., 2018; Nishida & Ochman, 2018). These results are consistent with a dietary shift towards anthropogenic food sources. The use of a single site per environment type is a limitation of our current study. Although we have previously demonstrated convergent environment‐related change in the grey squirrel microbiome between replicate city and exurban forest squirrel populations, this previous research was restricted to a narrower seasonal context (Stothart & Newman, 2021).

Diet is likely an important driver of the microbiome differences we observed in the eastern grey squirrel microbiome between urban and exurban environments, and across seasons. However, the resemblance of urban and exurban grey squirrel microbiomes in the autumn suggests that engrained animal behaviours (e.g., strong diet selection) might be able to maintain similarities in microbiome structure across disparate environments. Urban and exurban microbiome similarities in the autumn also suggest that inter‐environmental microbiome differences in wildlife populations might be over‐ or under‐estimated, depending on the seasonal context. Although many of the taxa we identified to significantly differ across seasons specialize on digesta, the greatest differential abundance estimates we observed were with respect to *Akkermansia*, a host mucus specialist. Future work should therefore seek to integrate dietary metabarcoding data, and comprehensive measures of host physiology to provide a more complete picture of seasonal rhythms in the host–microbiome relationship across urban and exurban landscapes.

## AUTHOR CONTRIBUTIONS

**Mason R. Stothart:** Conceptualization (equal); data curation (equal); formal analysis (equal); funding acquisition (supporting); investigation (lead); methodology (lead); writing – original draft (supporting); writing – review and editing (lead). **Hayley A. Spina:** Formal analysis (equal); writing – original draft (lead); writing – review and editing (supporting). **Michelle Z. Hotchkiss:** Conceptualization (supporting); data curation (supporting). **Winnie Ko:** Data curation (supporting). **Amy E. M. Newman:** Conceptualization (equal); project administration (equal); resources (lead); supervision (lead); writing – original draft (supporting); writing – review and editing (supporting).

### OPEN RESEARCH BADGES

This article has earned an Open Data badge for making publicly available the digitally‐shareable data necessary to reproduce the reported results. The data is available at [https://doi.org/10.5061/dryad.v6wwpzh2r].

## Supporting information


Appendix S1

Appendix S2

## Data Availability

Sample metadata and R scripts are available at https://doi.org/10.5061/dryad.v6wwpzh2r. Raw DNA sequences are available at NCBI SRA PRJNA701759.
