# Supplementary material for: Seasonal dynamics in the mammalian microbiome between disparate environments
Source: Ecol Evol. 2023 Dec 18;13(12):e10692. doi: 10.1002/ece3.10692 (PMC10726273; doi:10.1002/ece3.10692)
Supplement: Supplementary file 1 — Appendix S1 [file ECE3-13-e10692-s001.docx]

**Supplemental Information for:**

**Seasonal dynamics in the mammalian microbiome between disparate environments**

Stothart, M.R.^2^*, Spina, H.A.^1^*, Hotchkiss, M.Z.^3^, Ko, W.^1^, Newman, A.E.M.^1^

^1^Department of Integrative Biology, University of Guelph, Guelph, Ontario Canada

^2^Department of Ecosystem and Public Health, University of Calgary, Calgary Canada

^3^Department of Biology, University of Ottawa, Ontario Canada

*Authors contributed equally

**Table of Contents:**

| **Supplemental Table 1** | Page 2-4 |
| --- | --- |
| **Supplemental Tables 2-4** | Page 5 |
| **Supplemental Figure 1** | Page 6 |
| **Supplemental Figure 2** | Page 7 |

Supplemental Table 1: Meta-data corresponding to eastern grey squirrel fecal microbiome sequencing dataset.

| ID | Year | Month | Day | Season | Environment | Sex | Repro. Condition |
| --- | --- | --- | --- | --- | --- | --- | --- |
| F209 | 2017 | November | 1 | Autumn | Urban | F | NR |
| F202 | 2017 | November | 3 | Autumn | Urban | M | SCR |
| F226 | 2017 | November | 3 | Autumn | Urban | F | NR |
| F240 | 2017 | November | 3 | Autumn | Urban | M | SCR |
| F250 | 2017 | November | 3 | Autumn | Urban | F | NR |
| F210 | 2017 | November | 4 | Autumn | Urban | F | NR |
| F228 | 2017 | November | 4 | Autumn | Urban | M | SCR |
| F264 | 2017 | November | 4 | Autumn | Urban | F | NR |
| F270 | 2017 | November | 4 | Autumn | Urban | F | NR |
| F201 | 2017 | November | 12 | Autumn | Exurban | M | SCR |
| F212 | 2017 | November | 12 | Autumn | Exurban | F | NR |
| F237 | 2017 | November | 12 | Autumn | Exurban | F | NR |
| F249 | 2017 | November | 12 | Autumn | Exurban | M | SCR |
| F255 | 2017 | November | 12 | Autumn | Exurban | F | NR |
| F334 | 2017 | September | 4 | Autumn | Urban | M | NSCR |
| SC38 | 2018 | May | 1 | Spring | Urban | M | SCR |
| F160 | 2017 | May | 8 | Spring | Urban | M | SCR |
| F176 | 2017 | May | 8 | Spring | Urban | M | SCR |
| F103 | 2017 | May | 9 | Spring | Urban | F | NR |
| F168 | 2017 | May | 9 | Spring | Urban | F | NR |
| F174 | 2017 | May | 9 | Spring | Urban | M | SCR |
| F56 | 2017 | May | 9 | Spring | Urban | M | NSCR |
| F180 | 2017 | May | 10 | Spring | Urban | F | NR |
| F101 | 2017 | May | 11 | Spring | Urban | F | NR |
| F126 | 2017 | May | 12 | Spring | Urban | M | SCR |
| F152 | 2017 | May | 12 | Spring | Urban | M | SCR |
| F167 | 2017 | May | 12 | Spring | Urban | M | SCR |
| F182 | 2017 | May | 12 | Spring | Urban | F | NR |
| F92 | 2017 | May | 12 | Spring | Urban | F | NR |
| F137 | 2017 | May | 15 | Spring | Urban | F | NR |
| F141 | 2017 | May | 15 | Spring | Urban | F | NR |
| F150 | 2017 | May | 15 | Spring | Urban | F | NR |
| F161 | 2017 | May | 15 | Spring | Urban | F | NR |
| F170 | 2017 | May | 15 | Spring | Urban | F | NR |
| SC34 | 2018 | June | 5 | Spring | Exurban | M | SCR |
| SC29 | 2018 | June | 5 | Spring | Exurban | F | NR |
| SC37 | 2018 | June | 6 | Spring | Exurban | F | NR |
| SC31 | 2018 | June | 6 | Spring | Exurban | F | NR |
| SC35 | 2018 | June | 7 | Spring | Exurban | F | NR |
| SC32 | 2018 | June | 7 | Spring | Exurban | F | NR |
| SC33 | 2018 | June | 7 | Spring | Exurban | M | SCR |
| SC30 | 2018 | June | 8 | Spring | Exurban | F | NR |
| SC36 | 2018 | June | 8 | Spring | Exurban | F | NR |
| F34 | 2016 | June | 18 | Spring | Urban | F | NR |
| F49 | 2016 | June | 22 | Spring | Exurban | F | NR |
| F53 | 2016 | June | 22 | Spring | Exurban | M | SCR |
| F44 | 2016 | June | 23 | Spring | Exurban | F | NR |
| F47 | 2016 | June | 23 | Spring | Exurban | F | NR |
| F59 | 2016 | June | 23 | Spring | Exurban | M | SCR |
| F50 | 2016 | June | 24 | Spring | Exurban | F | NR |
| F20 | 2016 | August | 4 | Summer | Urban | M | NSCR |
| F96 | 2016 | August | 10 | Summer | Urban | M | NSCR |
| F63 | 2016 | August | 11 | Summer | Urban | M | NSCR |
| F90 | 2016 | August | 11 | Summer | Urban | M | NSCR |
| F61b | 2017 | July | 3 | Summer | Urban | M | SCR |
| F33 | 2017 | July | 4 | Summer | Urban | M | SCR |
| F64b | 2017 | July | 4 | Summer | Urban | M | SCR |
| F66 | 2016 | July | 5 | Summer | Urban | F | NR |
| F72 | 2016 | July | 5 | Summer | Urban | F | NR |
| F61 | 2016 | July | 7 | Summer | Urban | F | NR |
| F68 | 2016 | July | 7 | Summer | Urban | F | NR |
| F171 | 2017 | July | 9 | Summer | Exurban | M | SCR |
| F179 | 2017 | July | 9 | Summer | Exurban | M | SCR |
| F182b | 2017 | July | 9 | Summer | Exurban | M | SCR |
| F94 | 2017 | July | 9 | Summer | Exurban | M | SCR |
| F97b | 2017 | July | 9 | Summer | Exurban | F | R |
| F183 | 2017 | July | 11 | Summer | Exurban | F | R |
| F194 | 2017 | July | 11 | Summer | Exurban | F | R |
| F97 | 2016 | July | 12 | Summer | Exurban | M | SCR |
| F99 | 2016 | July | 12 | Summer | Exurban | M | NSCR |
| F173 | 2017 | July | 12 | Summer | Exurban | M | SCR |
| F83 | 2017 | July | 12 | Summer | Exurban | M | SCR |
| F93 | 2017 | July | 12 | Summer | Exurban | M | SCR |
| F143 | 2017 | July | 14 | Summer | Exurban | M | SCR |
| F186 | 2017 | July | 14 | Summer | Exurban | M | SCR |
| F104 | 2016 | July | 15 | Summer | Exurban | F | NR |
| F64 | 2016 | July | 15 | Summer | Exurban | M | NSCR |
| F76 | 2016 | July | 15 | Summer | Exurban | F | NR |
| F67 | 2016 | July | 19 | Summer | Exurban | M | NSCR |
| F88 | 2016 | July | 19 | Summer | Exurban | M | NSCR |
| F98 | 2016 | July | 19 | Summer | Exurban | M | NSCR |
| F166 | 2017 | July | 19 | Summer | Urban | F | NR |
| F1 | 2017 | July | 20 | Summer | Urban | F | NR |
| F174b | 2017 | July | 20 | Summer | Urban | F | NR |
| F197 | 2017 | July | 20 | Summer | Urban | M | SCR |
| F184 | 2017 | July | 26 | Summer | Urban | F | NR |
| F198 | 2017 | July | 26 | Summer | Urban | F | NR |
| SC1 | 2018 | Feb | 10 | Winter | Urban | F | NR |
| SC2 | 2018 | Feb | 10 | Winter | Urban | M | SCR |
| SC4 | 2018 | Feb | 10 | Winter | Urban | F | R |
| SC3 | 2018 | Feb | 10 | Winter | Urban | F | R |
| SC6 | 2018 | Feb | 12 | Winter | Urban | F | R |
| SC5 | 2018 | Feb | 12 | Winter | Urban | F | NR |
| SC7 | 2018 | Feb | 13 | Winter | Urban | M | SCR |
| SC10 | 2018 | Feb | 13 | Winter | Urban | F | R |
| SC9 | 2018 | Feb | 13 | Winter | Urban | F | R |
| SC8 | 2018 | Feb | 13 | Winter | Urban | F | R |
| SC12 | 2018 | Feb | 14 | Winter | Urban | M | SCR |
| SC14 | 2018 | Feb | 14 | Winter | Urban | M | SCR |
| SC15 | 2018 | Feb | 14 | Winter | Urban | F | NR |
| SC13 | 2018 | Feb | 14 | Winter | Urban | M | SCR |
| SC17 | 2018 | Feb | 14 | Winter | Urban | M | SCR |
| SC18 | 2018 | Feb | 14 | Winter | Urban | F | R |
| SC11 | 2018 | Feb | 14 | Winter | Urban | M | SCR |
| SC21 | 2018 | Feb | 18 | Winter | Exurban | M | SCR |
| SC25 | 2018 | Feb | 18 | Winter | Exurban | M | SCR |
| SC23 | 2018 | Feb | 18 | Winter | Exurban | F | NR |
| SC20 | 2018 | Feb | 18 | Winter | Exurban | M | SCR |
| SC24 | 2018 | Feb | 22 | Winter | Exurban | M | SCR |
| SC26 | 2018 | Feb | 23 | Winter | Exurban | M | SCR |
| SC27 | 2018 | Feb | 23 | Winter | Exurban | F | NR |
| SC28 | 2018 | Feb | 23 | Winter | Exurban | M | SCR |

Supplemental Table 2: PERMANOVA results of pairwise seasonal comparisons of eastern grey squirrel bacterial microbiome Bray-Curtis dissimilarities matrices. * denotes significance at p < 0.05.

|  | **All** | | | | |
| --- | --- | --- | --- | --- | --- |
| **Term** | | **N** | **R^2^** | **F** | **P value** |
| Summer v. Spring | | 72 | 0.03 | 1.96 | <0.01* |
| Summer v. Autumn | | 52 | 0.06 | 3.23 | <0.01* |
| Summer v. Winter | | 62 | 0.04 | 2.52 | <0.01* |
| Spring v. Autumn | | 50 | 0.06 | 2.91 | <0.01* |
| Spring v. Winter | | 60 | 0.03 | 1.86 | <0.01* |
| Winter v. Autumn | | 40 | 0.05 | 2.02 | <0.01* |

Supplemental Table 3: PERMANOVA results of pairwise seasonal comparisons of urban eastern grey squirrel bacterial microbiome Bray-Curtis dissimilarities matrices. * denotes significance at p < 0.05.

|  | **Urban** | | | | |
| --- | --- | --- | --- | --- | --- |
| **Term** | | **N** | **R^2^** | **F** | **P value** |
| Summer v. Spring | | 37 | 0.04 | 1.37 | 0.04* |
| Summer v. Autumn | | 27 | 0.09 | 2.59 | <0.01* |
| Summer v. Winter | | 34 | 0.05 | 1.80 | <0.01* |
| Spring v. Autumn | | 30 | 0.09 | 2.73 | <0.01* |
| Spring v. Winter | | 37 | 0.05 | 1.72 | <0.01* |
| Winter v. Autumn | | 27 | 0.08 | 2.20 | <0.01* |

Supplemental Table 4: PERMANOVA results of pairwise seasonal comparisons of exurban eastern grey squirrel bacterial microbiome Bray-Curtis dissimilarities matrices. * denotes significance at p < 0.05.

|  | **Exurban** | | | | |
| --- | --- | --- | --- | --- | --- |
| **Term** | | **N** | **R^2^** | **F** | **P value** |
| Summer v. Spring | | 25 | 0.07 | 1.80 | <0.01* |
| Summer v. Autumn | | 35 | 0.05 | 1.86 | <0.01* |
| Summer v. Winter | | 28 | 0.08 | 2.41 | <0.01* |
| Spring v. Autumn | | 20 | 0.08 | 1.55 | <0.01* |
| Spring v. Winter | | 23 | 0.09 | 1.96 | <0.01* |
| Winter v. Autumn | | 13 | 0.09 | 1.10 | 0.29 |


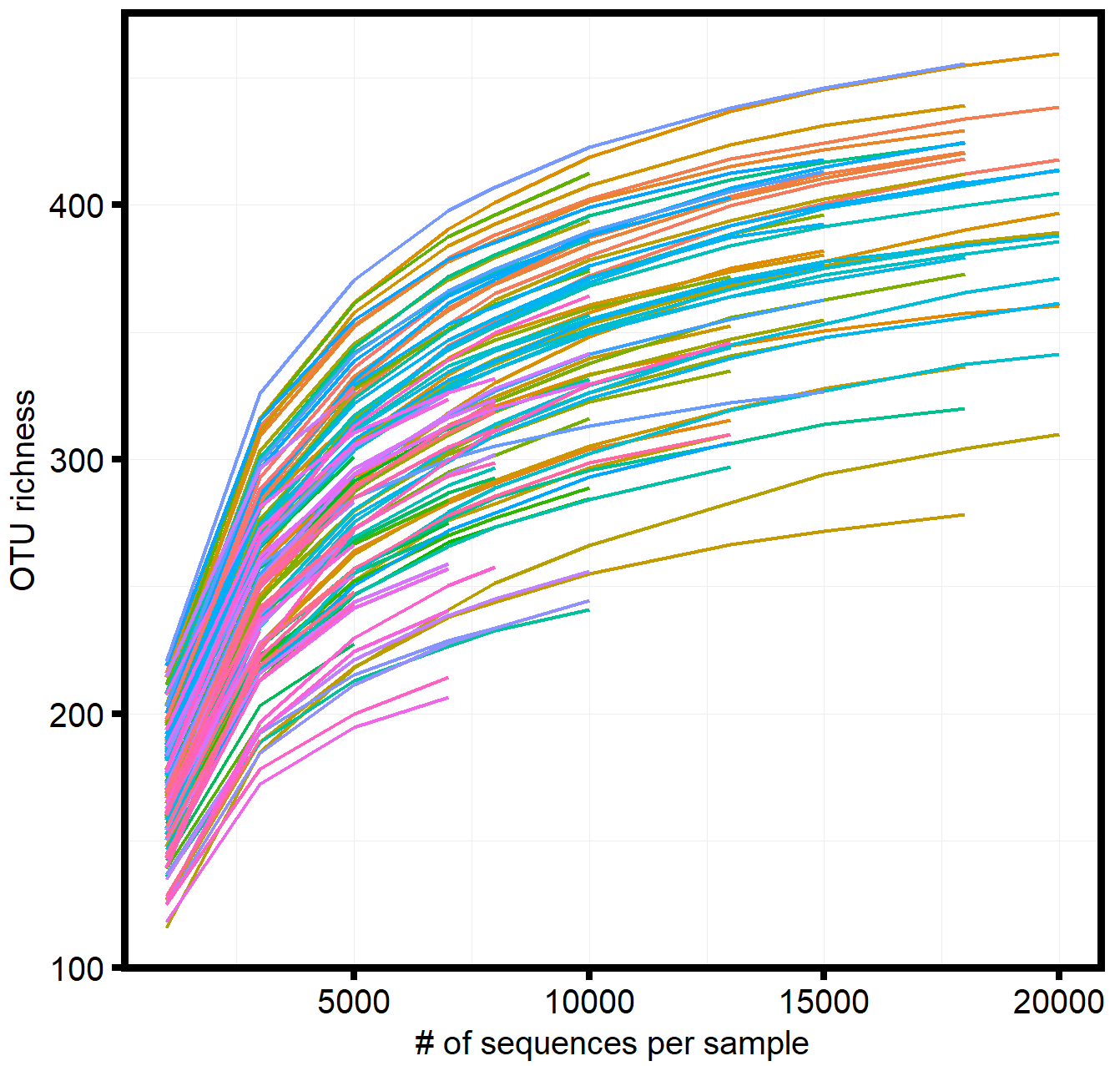


Supplemental Figure 1: Rarefaction curve of OTU richness in the eastern grey squirrel fecal microbiome. Each line corresponds to a different sample.


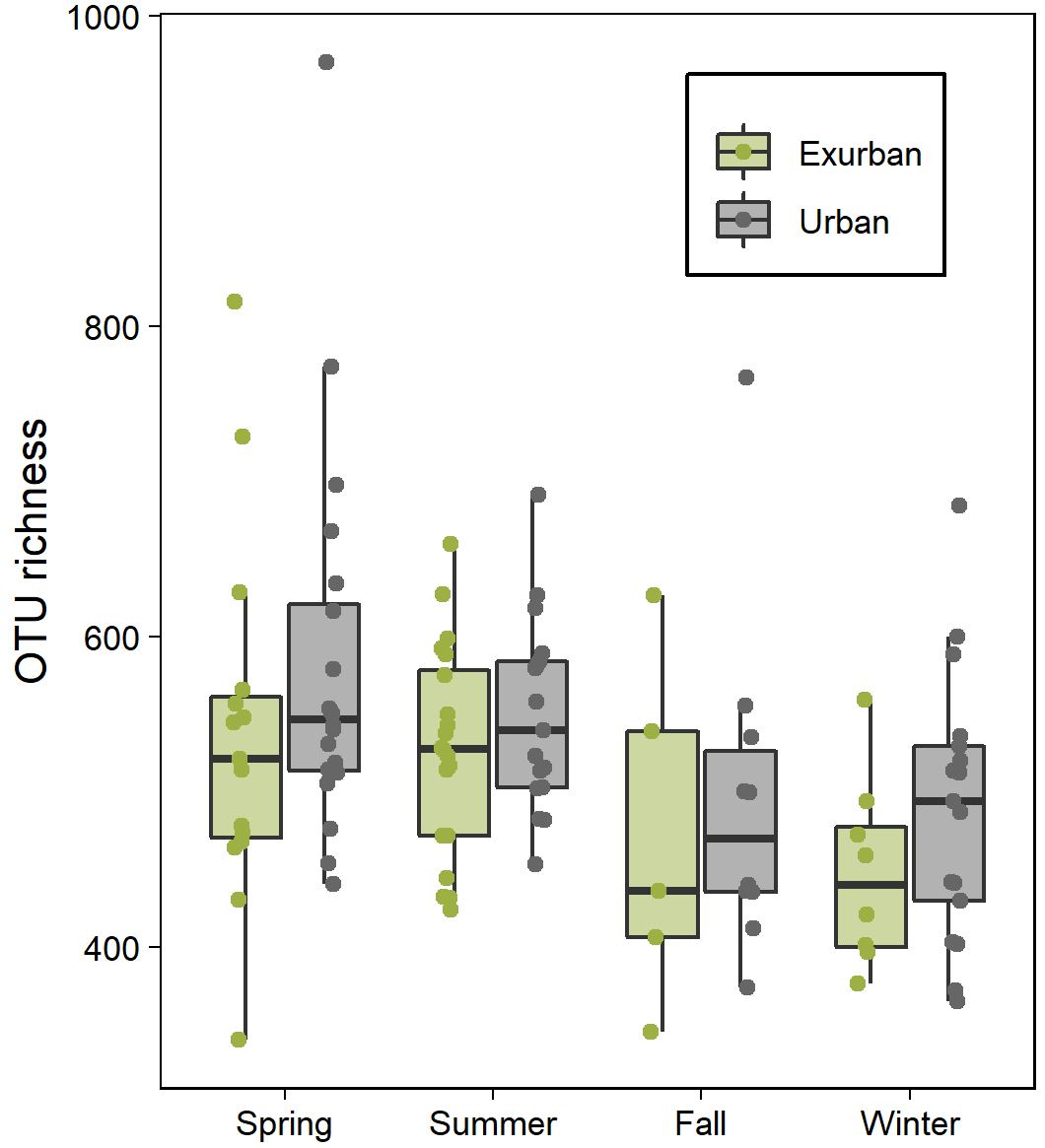


Supplemental Figure 2: Boxplot of OTU richness in the eastern grey squirrel fecal microbiome separated by season and environment. Estimates of OTU richness derived from the ‘breakaway’ R package.
